# Cardiovascular disease risk profile and microvascular complications of diabetes: comparison of Indigenous cohorts with diabetes in Australia and Canada

**DOI:** 10.1186/1475-2840-11-30

**Published:** 2012-03-28

**Authors:** Louise J Maple-Brown, Joan Cunningham, Bernard Zinman, Mary Mamakeesick, Stewart B Harris, Philip W Connelly, Jonathan Shaw, Kerin O'Dea, Anthony J Hanley

**Affiliations:** 1 Menzies School of Health Research, Charles Darwin University, Darwin, NT, Australia; 2 Division of Medicine, Royal Darwin Hospital, Darwin, NT, Australia; 3 Leadership Sinai Centre for Diabetes, Mt Sinai Hospital, Toronto, Canada; 4 Division of Endocrinology, University of Toronto, Toronto, Canada; 5 Sandy Lake Health and Diabetes Project, Sandy Lake First Nation, London, ON, Canada; 6 Centre for Studies in Family Medicine at The Schulich School of Medicine and Dentistry, University of Western Ontario, London, ON, Canada; 7 Keenan Research Centre in the Li Ka Shing Knowledge Institute of St Michael's Hospital, Toronto, Canada; 8 Department of Laboratory Medicine and Pathobiology, University of Toronto, Toronto, Canada; 9 Baker IDI Heart and Diabetes Institute, Melbourne, Australia; 10 Sansom Institute for Health Research, University of South Australia, Adelaide, South, Australia; 11 Department of Nutritional Sciences and Dalla Lana School of Public Health, University of Toronto, Toronto, Canada

**Keywords:** Aboriginal, Retinopathy, Albuminuria, Peripheral neuropathy, Perhipheral vascular disease

## Abstract

**Background:**

Indigenous populations of Australia and Canada experience disproportionately high rates of chronic disease. Our goal was to compare cardiovascular (CVD) risk profile and diabetes complications from three recent comprehensive studies of diabetes complications in different Indigenous populations in Australia and Canada.

**Methods:**

We compared participants from three recent studies: remote Indigenous Australians (2002-2003, n = 37 known diabetes), urban Indigenous Australians (2003-2005, n = 99 known diabetes), and remote Aboriginal Canadians (2001-2002, n = 188 known diabetes).

**Results:**

The three groups were similar for HbA1c, systolic BP, diabetes duration. Although leaner by body-mass-index criteria, remote Indigenous Australians displayed a more adverse CVD risk profile with respect to: waist-hip-ratio (1.03, 0.99, 0.94, remote Indigenous Australians, urban Indigenous Australians, remote Canadians, p < 0.001); HDL-cholesterol (0.82, 0.96, 1.17 mmol/L, p < 0.001); urine albumin-creatinine-ratio (10.3, 2.4, 4.5 mg/mmol); and C-reactive protein. With respect to diabetes complications, microalbuminuria (50%, 25%, 41%, p = 0.001) was more common among both remote groups than urban Indigenous Australians, but there were no differences for peripheral neuropathy, retinopathy or peripheral vascular disease.

**Conclusions:**

Although there are many similarities in diabetes phenotype in Indigenous populations, this comparison demonstrates that CVD risk profiles and diabetes complications may differ among groups. Irrespective, management and intervention strategies are required from a young age in Indigenous populations and need to be designed in consultation with communities and tailored to community and individual needs.

## Background

The burden of type 2 diabetes, chronic kidney disease (CKD) and cardiovascular disease (CVD) has had devastating effects on Indigenous populations of both Australia and Canada, and there appear to be close similarities in the disease presentation and impact between the Indigenous populations of these geographically disparate continents. Both populations have earlier onset of chronic conditions such as diabetes and CVD compared to the non-Indigenous population, and chronic conditions are the greatest single contributor to the premature mortality in both Indigenous populations [1-5].

Despite the high prevalence of diabetes, CVD and CKD among Indigenous populations globally, there is little published data from studies using detailed participant examinations for assessment of complications of diabetes [6,7]. Diabetic nephropathy has been well described in both Indigenous Australians and Canadians [8,9], but there are fewer available data on retinopathy, neuropathy and peripheral vascular disease (PVD). CVD is the leading cause of premature mortality among Indigenous Australians and Canadians [1,4,5,10], and rates of CVD and of CVD-related mortality appear to be increasing among these populations, despite a reduction in the non-Indigenous populations of both nations [11-13].

In light of these apparent similarities in early onset and high prevalence of both type 2 diabetes and CVD among the Indigenous populations of Australia and Canada, we aimed to compare cardiovascular risk profile and microvascular complications of diabetes from three recent comprehensive, although small clinical studies of Aboriginal cohorts with diabetes from Australia and Canada: 1) a remote Aboriginal community in Northeast Arnhemland, Northern Territory, Australia; 2) the Darwin Region Urban Indigenous Diabetes (DRUID) Study, Northern Territory, Australia; and 3) the Sandy Lake Diabetes Complications Study, from a remote First Nations community from northwestern Ontario, Canada [14-16].

## Methods

### Participants

Methods for the three studies have been described previously [14,15,17-19]. In brief, the Australian remote Aboriginal community is on an island approximately 550 km from Darwin (the capital city of the Northern Territory) and has a centralised population of approximately 1500. This was the first community-wide screening for risk of diabetes and CVD to be conducted in this community. The protocol was approved by the Joint Human Research Ethics Committee (HREC) of the Northern Territory Department of Health and Community Services and Menzies School of Health Research, which includes an Aboriginal sub-committee with the right of veto for studies involving Indigenous Australians. The Community Council endorsed the study in June 2001. The study was conducted from August 2001 to March 2002, with participants with diabetes returning for assessment of diabetes complications in March-May 2002. Participants were aged 15 years and over. Anthropometric measurements and fasting blood samples were collected from 332 of the eligible 706 residents aged 15 and over; 40 participants (12%) were diagnosed with diabetes. At the time of the study, health care was delivered by a community-controlled health organisation (funded by the federal government), staffed by one resident general practitioner, remote area nurses and Aboriginal Health Workers, with visiting outreach specialist services from Darwin (a 3 hour flight away).

The DRUID Study was a volunteer cohort of approximately 1000 urban Indigenous people from Darwin, Australia, undertaken from September 2003 to March 2005. Darwin is a tropical port city of approximately 100,000 people located on the northern coast of Australia. Participants met the following eligibility criteria: identified as Aboriginal or Torres Strait Islander; aged ≥ 15 years; had resided within a specified geographical region around Darwin for at least 6 months; and were living in a private dwelling. The DRUID cohort represented approximately 14% of the estimated target population, and therefore is not necessarily representative. The DRUID study represents a heterogeneous and diverse population group with significant ethnic admixture (with ancestry including Aboriginal, Torres Strait Islander, and a range of European and Asian ethnicities), diversity in language, and participants from a broad range of social and economic circumstances, from impoverished people living in insecure housing to senior public servants and business leaders. Participants ranged from those who had lived in Darwin all their lives to those who had relocated from a remote Aboriginal community to Darwin within recent years (but not in the previous 6 months). Participants accessed a variety of primary health care providers, from private general practitioners (45%), to Aboriginal health services (32%) to nil (11%) [20]. Of the 99 participants with known diabetes reported here: 87% were of Aboriginal background, 7% were of Torres Strait Islander background and 6% reported both Aboriginal and Torres Strait Islander background; 50% reported at least one non-Indigenous grandparent. The DRUID study was approved by the same HREC as the remote Australian study.

The Canadian study was The Sandy Lake Diabetes Complications Study, implemented as part of the Sandy Lake Health and Diabetes Project, a population-based cohort study involving participants aged 10 years and over, with collection of baseline data from 1993-1995 [21]. Crude diabetes prevalence was 17.2% in the baseline survey (which involved 728 participants, 72% of the eligible population). The Sandy Lake Diabetes Complications Study involved community members with type 2 diabetes and was performed from September 2001 to July 2002, with approval from the Sandy Lake First Nation Band Council and University of Toronto Ethics Review Committee. The community of Sandy Lake, Ontario, is located about 2000 km northwest of Toronto, in the subarctic boreal forest region of central Canada, with a population of approximately 2300 Oji-Cree. Health care is delivered at a nursing station operated by the federal government and staffed by outpost nurse practitioners. The history of the community is similar to that of the remote Australian community in that people of Sandy Lake previously led a hunter-gatherer life in small, extended family groups, with rapid lifestyle changes beginning mid-twentieth century [22].

### Diagnosis of diabetes

For consistency across the three study groups, only those with a previous diabetes diagnosis are included here, not those newly diagnosed by the Australian studies. Remote Australian study: previous diabetes diagnosis confirmed from medical records, n = 37 (100% of diabetic patients known to the clinic and currently living in the community). Urban Australian Study: participants classified as having known diabetes if a diagnosis was self-reported and they were either on current diabetes medication or their glucose values on 75 g oral glucose tolerance test (OGTT) met the 1999 WHO criteria for diabetes [23]. Both participants known to have diabetes and those newly diagnosed with diabetes by the study were invited to return to participate in additional tests for assessment of diabetic complications, 79% of eligible participants completed the complications assessment, which was performed within 3 months of baseline assessment for 79%, within 6 months for 93%. Only those with previously diagnosed diabetes are presented here (n = 99). Remote Canadian Study: all members of the Sandy Lake community with type 2 diabetes were invited to participate in the study. Several data sources were used to identify individuals, including health clinic records and records of the baseline research study (in which OGTTs were used to diagnose diabetes). A total of 188 of 250 (75%) eligible participants with diabetes consented to the complications study [16].

### Assessment of diabetes complications

The full protocols for assessment of diabetes complications have been previously described [14-16,18,19]. There were some differences in methods between studies for anthropometry, blood pressure, biochemistry and assessment of diabetes complications (Table 1). Regarding nephropathy, creatinine was not assessed in the remote Australian study and was measured in the other studies prior to introduction of creatinine assays traceable to the international reference method of Isotope Dilution Mass Spectrometry (IDMS), thus eGFR was calculated using the "186" MDRD (modification of diet in renal disease) formula (appropriate for creatinine measurements not calibrated to IDMS). Urine albumin-creatinine ratio (ACR) was assessed at the point-of-care by DCA2000 (Bayer Diagnostics, Tarrytown, NY, USA) in the remote Canadian study, which has been validated against laboratory techniques (used in the Australian studies) [24]. Microalbuminuria was defined as urine ACR ≥ 2.5 and ≤ 25 mg/mmol in men and ≥ 3.5 and ≤ 25 mg/mmol in women. Macroalbuminuria was defined as ACR > 25 mg/mmol [25]. There were considerable differences between studies for methods of neuropathy assessment. Although the definition used in previous publications for the two Australian studies differed slightly, comparable data was collected by both studies thus the definition of neuropathy for the remote Australian study could be revised to match that of urban study. However as this was not possible for the Canadian study, rates of neuropathy presented are as published for each study previously. As use of 10 gm monofilament is consistent across all studies, results are presented in addition to that of the original peripheral neuropathy assessment. Retinopathy was assessed by digital retinal photography in both the urban Australian and remote Canadian studies but by chart review in the remote Australian study. All 3 studies utilised ankle brachial pressure index (ABPI) and standardised claudication questionnaires for assessment of PVD. PVD was defined as present if ABPI < 0.9 and/or claudication symptoms were present (in all groups for the purposes of this comparison, although the ABPI cut-point used differed in previous publications for each study).

**Table 1:** Comparison of methods in the three studies

Measure	Remote Australian	Urban Australian	Remote Canadian
Dates	2002-2003	2003-2005	2001-2002

Waist	Measured at the point midway between the lowest rib margin and iliac crest, using a non-stretch tape measure in a horizontal plane at the end of expiration	Measured at the point midway between the lowest rib margin and iliac crest, using a non-stretch tape measure in a horizontal plane at the end of expiration	Measured at the natural indentation between the umbilicus and xiphoid process as viewed from behind; using an inelastic tape in horizontal plane over light clothing

Hip	Measured at the point yielding the maximum circumference over the buttocks with the tape in a horizontal plane over very light clothing	Measured at the point yielding the maximum circumference over the buttocks with the tape in a horizontal plane over very light clothing	Measured at the point of maximum extension of the buttocks as viewed from the side; using an inelastic tape in horizontal plane over light clothing

Weight	Digital portable scale	Seca digital portable scale (Model 767, Seca Deutschland, Hamburg, Germany)	Balance beam scale (Health-o-meter Inc, Bridgeview, USA)

Blood pressure & pulse	Automated Dinamap (Critikon XL, Johnson and Johnson, USA)	Welch Allyn Spot Vital signs monitor (Welch Allyn Medical Products, Skaneateles Falls, USA)	Hand-held aneroid sphygmomanometer

Lipids	Hitachi 917 (standard automated colorimetric methods using commercial reagents)	Hitachi 917 enzymatic	Dextran sulfate - magnesium precipitation of non-HDL cholesterol. Technicon RA-1000 and Technicon reagents for measurement of cholesterol and triglyceride

HbA1c	Cation exchange high performance liquid chromatography (HPLC) on a Pharmacia MonoS column (results traceable to DCCT method)	Cation exchange high performance liquid chromatography (HPLC) on a Pharmacia MonoS column (results traceable to DCCT method)	Bayer DCA point-of-care

Serum creatinine	Not performed	Kinetic Jaffe (Hitachi 917), prior to introduction of creatinine assays traceable to IDMS	Jaffe (Beckman Coulter Synchron LX20), prior to introduction of creatinine assays traceable to IDMS

Urine albumin	Immunonephelometry (Beckman array 360)	Immunonephelometry (Beckman array 360)	Bayer DCA point-of-care

Urine creatinine	Kinetic Jaffe (Hitachi 917)	Kinetic Jaffe (Hitachi 917)	Bayer DCA point-of-care

Homocys-teine	HPLC after conversion to a fluorescent derivative with ABDF (7-Fluoro-2,1,3-benzoxadiazole-4-sulfonamide)	HPLC after conversion to a fluorescent derivative with ABDF (7-Fluoro-2,1,3-benzoxadiazole-4-sulfonamide)	Abbot IMx

CRP	High sensitivity commercial assay (BN-II Nephelometer; Dade Behring Diagnostics, Lane Cove NSW; intra and inter assay CVs of 1.9% and 4.4% respectively, with a lower limit of detection of 0.16 mg/L).	High sensitivity, immunoturbidimetry (Hitachi 917)	High sensitivity, Dade-Behring, BN-100 Nephelometer.

Neuropathy	Peripheral neuropathy was defined as present if two or more of the following were abnormal: pressure perception test (monofilament), vibration sensation (128 Hz tuning fork), pain sensation, temperature sensation, ankle deep tendon reflexes [14]	Peripheral neuropathy was defined as present if two or more of the following were abnormal: modified neuropathy symptom score; modified neuropathy disability score; pressure perception test; fall in systolic blood pressure of ≥ 20 mmHg after standing for 60s [15]	Modified Michigan Neuropathy Screening Instrument (modification involved addition of monofilament and removal of questionnaire component). Scores range from 0 to 9, and neuropathy defined as present if score > 2.

Retinopathy	Determined from chart review	Digital retinal photographs, Topcon non-mydriatic retinal camera (Model TRC-NW5, Topcon Corporation, Tokyo, Japan). Images graded by a single ophthalmologist at the Centre for Eye Research Australia (Melbourne, Australia) according to simplified Wisconsin scale: no diabetic retinopathy (DR), mild or moderate non-proliferative DR (NPDR), severe NPDR or proliferative DR, fundus pathology other than DR. Retinopathy includes NPDR or PDR in 1 or both eyes.	Digital retinal photographs, Topcon non-mydriatic retinal camera (Model TRC-NW100, Topcon Canada, Waterloo, Ontario, Canada). Images graded at the Ocular Epidemiology Grading Centre at the University of Wisconsin according to scale of mild NPDR, moderate or severe NPDR, proliferative DR or macula edema.

PVD: Ankle Brachial Pressure Index	Doppler (Hadeco Bidirectional Smartdop 20; Hayashi Denki Co. Ltd., Kawasaki, Japan) and manual anaeroid sphygmomanometer (Welch Allyn Medical Products, Skaneateles Falls, USA)	Doppler probe (Dopplex High Sensitivity Pocket Doppler Model D900, Huntleigh Healthcare Pty Ltd, Hamilton Hill, WA, Australia) and manual anaeroid sphygmomanometer (Welch Allyn Medical Products, Skaneateles Falls, USA)	Doppler stethoscope and manual anaeroid sphygmomanometer.

### Statistical analysis

Data analysis was performed using Stata version 10.1 (Stata Corporation, TX, USA). Variables with distributions significantly different from normal were log transformed. Data are presented as frequency (%), mean (S.D.) or geometric mean (95% confidence interval). To determine associations between variables across study groups, Pearson chi-square tests (categorical variables) or independent sample t-tests (continuous variables) were conducted in the whole group as well as stratified by gender, due to differences between groups in proportion of female participants. Median duration of diabetes was compared between groups on pair-wise comparison using the Kruskal-Wallis test. Multiple regression analysis of albuminuria was performed using the backwards selection method, for groups combined and stratified by study. Established risk factors and variables with p < 0.05 on univariate analysis were considered for entry into the model. Model fit was assessed using likelihood ratio-chi square. Statistical significance was accepted at p < 0.05.

## Results

A comparison of the characteristics of diabetic participants of the three study groups is presented in Table 2. Rates of current cigarette smoking were higher in both remote Indigenous Australians and Canadians compared to urban Indigenous Australians. Duration of diabetes was not significantly different across groups. Remote Indigenous Australian participants had significantly lower body mass index (BMI) than the other groups but higher waist-hip ratio (WHR), due to lower hip circumference. The remote Canadian group also displayed significantly lower BMI and waist-hip ratio compared to the urban Indigenous Australian group. On stratification by gender, differences between urban Australian and remote Canadian participants for height (both genders) and BMI (in women) were no longer significant, but differences in waist and WHR remained significant in both genders (higher in urban Australian participants). Differences between urban and remote Australian participants remained significant for all anthropometric variables in women and for weight, BMI, waist, hip (but not height and WHR) in men. Results were similar when adjusted for age and sex (data not shown).

**Table 2:** Characteristics of participants with known diabetes across the 3 studies

	Group 1: Remote Australian (n = 37)	Group 2: Urban Australian (n = 99)	Group 3: Remote Canadian (n = 188)	p values		
				
				Group 1 vs 2	Group 1 vs 3	Group 2 vs 3
**Age **(years)	51 ± 8	53 ± 10	47 ± 13	0.25	0.078	< 0.001

**Female Sex**^§^	20 (54%)	75 (76%)	113 (60%)	0.014	0.494	0.008

**Current Smoker**^§^	17 (49%)	22 (26%)	93 (51%)	0.016	0.784	< 0.001

**Diabetes Duration **(years)*	6 (1 - 15)	7.5 (0 - 36)	9 (0 - 42)	0.144	0.114	0.643

**Weight **(kg)	76.3 ± 14.0	86.4 ± 17.2	84.1 ± 16.7	0.002	0.008	0.279

**Height **(cm)	168.6 ± 8.5	163.4 ± 8.4	166.5 ± 8.6	0.002	0.180	0.004

**BMI **(kg/m^2^)	26.7 ± 3.8	32.4 ± 6.1	30.2 ± 5.3	< 0.001	< 0.001	0.003

**Waist **(cm)	101.0 ± 9.7	108.8 ± 12.6	103.5 ± 10.8	0.001	0.199	< 0.001

**Hip **(cm)	98.0 ± 8.0	110.6 ± 13.4	110.5 ± 11.5	< 0.001	< 0.001	0.968

**WHR**	1.03 ± 0.07	0.99 ± 0.09	0.94 ± 0.07	0.014	< 0.001	< 0.001

**Heart Rate **(bpm)	76 ± 11	76 ± 11	71 ± 10	0.820	0.021	< 0.001

**Systolic BP**(mmHg)	131 ± 26	128 ± 18	125 ± 17	0.408	0.065	0.183

**Diastol BP**(mmHg)	76 ± 11	78 ± 10	74 ± 9	0.2112	0.315	< 0.001

**HbA1c**^† ^(%)	8.7 (7.8-9.6)	8.1 (7.7 - 8.4)	8.0 (7.7 - 8.3)	0.123	0.111	0.751

**Total cholesterol**^† ^(mmol/L)	4.8 (4.5-5.0)	5.0 (4.8-5.3)	4.8 (4.6 - 4.9)	0.253	0.972	0.061

**Triglycerides**^† ^(mmol/L)	2.4 (2.0-2.8)	2.4 (2.1 - 2.8)	1.7 (1.6 - 1.9)	0.935	< 0.001	< 0.001

**HDL-cholesterol**^† ^(mmol/L)	0.82 (0.76-0.89)	0.96 (0.91-1.02)	1.17 (1.11-1.23)	0.006	< 0.001	< 0.001

**Creatinine**^† ^(umol/L)	-	72.7 (67.8-77.9)	62.8 (60.1-65.8)	-	-	< 0.001

**eGFR (MDRD)**	-	83.6 (77.6-90.1)	107.1 (102-112)	-	-	< 0.001

**CRP**^† ^(mg/L)	6.6 (4.7 - 9.3)	6.8 (5.7 - 8.1)	3.1 (2.6 - 3.6)	0.870	< 0.001	< 0.001

**Homocysteine**^† ^(umol/L)	12.4(10.8-14)	10.2 (9.5-11.1)	7.1 (6.8 - 7.5)	0.016	< 0.001	< 0.001

**Urine ACR**^† ^(mg/mmol)	10.3(5.7-18.5)	2.4 (1.6-3.8)	4.5 (3.6 - 5.6)	0.004	0.003	0.007

Data are mean ± standard deviation unless stated otherwise. Biochemistry is fasting.

**^§^**Data are n(%). *Data are median (range). **^†^**Data are geometric mean (95% confidence interval).

BMI, body mass index; WHR, waist-hip ratio; BP, blood pressure; Diastol, diastolic; eGFR (MDRD), estimated glomerular filtration rate (Modification of Diet in Renal Disease formula); CRP, C-reactive protein; ACR, albumin-creatinine ratio.

Remote Canadian participants displayed a significantly lower diastolic blood pressure than urban Indigenous Australians and lower heart rate than both Australian groups (Table 2), with differences remaining significant on stratification by gender (except for male urban Australians compared to remote Canadians).

The remote Australian group had significantly lower HDL-cholesterol and higher urine ACR than both urban Australians and remote Canadians. Of note, compared to each of the Australian groups, the remote Canadian group demonstrated higher HDL-cholesterol and lower triglycerides, C-reactive protein (CRP) and homocysteine. However, compared to urban Indigenous Australians, remote Indigenous Canadians had higher urine ACR (but better kidney function as determined by MDRD formula for eGFR). Differences between urban Australian and remote Canadian participants remained for all biochemical variables on stratification by gender with the exception of ACR for women.

A comparison of current diabetes management is presented in Table 3. The notable difference between the groups is use of insulin, which was not used for any remote Australian participants but was used to a similar extent in both urban Indigenous Australians and remote Canadians. Metformin was used more frequently in both Australian groups than in the remote Canadian group. Use of angiotensin converting enzyme inhibitors or angiotensin 2 receptor antagonists was similarly high in all groups. Of participants with micro or macro-albuminuria, 80%, 73% and 73% respectively of remote Australians, urban Australians, remote Canadians were treated with angiotensin converting enzyme inhibitors or angiotensin 2 receptor antagonists.

**Table 3:** Diabetes management in urban and remote Indigenous Australians and remote Indigenous Canadians

	Group 1: Remote Australian (n = 37)	Group 2: Urban Australian (n = 94)	Group 3: Remote Canadian (n = 188)
Diet/nil glucose-lowering medications	11 (30%)	9 (10%)	78 (41%)
Metformin	24 (65%)	52 (57%)	83 (44%)^†^‡
Sulphonylurea	11 (30%)	32 (35%)	62 (33%)
Acarbose	0	2 (2%)	1 (0.5%)
Thiazolidinedione	0	6 (7%)	5 (3%)
Insulin	0	18 (19%)^†^	34 (18%)^†^
Aspirin*	16 (43%)	39 (43%)	36 (32%)
HMG CoA reductase inhibitor*	9 (24%)	35 (37%)	23 (20%)‡
ACE inhibitor or A2RA*	23 (62%)	59 (63%)	73 (64%)

Data are n (%).

^† ^p < 0.0001 when compared to Group 1

‡ p < 0.05 compared to Group 2

*n = 114 for Remote Canadian group

HMG CoA, 3-hydroxy-3-methylglutaryl-CoA; ACE, angiotensin converting enzyme; A2RA, angiotensin-2 receptor antagonist.

Table 4 describes the frequency of complications in the three groups. Rates of microalbuminuria were higher in both remote groups compared to the urban group. Macroalbuminuria was similar for remote Canadians and urban Indigenous Australians but higher for remote Indigenous Australians. Both remote groups had greater rates of neuropathy than the urban Indigenous group, with the highest rate in the remote Canadian group (but there were methodological differences between all 3 studies). When assessment was based on results of the pressure perception test (the common neuropathy test across the studies), there were no significant differences in neuropathy between any of the groups; although rates appeared to be higher in both remote groups than the urban group, this difference was not significant. There were no significant differences in rates of retinopathy or PVD between the 3 groups, nor for rates of current foot ulcer or previous lower extremity amputation. It was notable that fewer remote Australian participants reported usually wearing footwear than urban Australian participants: 25% compared to 90%, p < 0.001. Logistic regression (Table 5) revealed that rates of albuminuria were independently higher in both remote Australians and remote Canadians compared to urban Australians with diabetes, after adjustment for systolic blood pressure, HbA1c and diabetes duration. On stratification by study group, determinants of albuminuria were similar in each of the three groups: systolic blood pressure was a significant determinant in each study group, while diabetes duration was an additional determinant in urban Australians and remote Canadians, and HbA1c significant in remote Australians (data not shown).

**Table 4:** Complications of diabetes in urban and remote Indigenous Australians and remote Indigenous Canadians

Complication	Group 1: Remote Australian (n = 36*)	Group 2: Urban Australian (n = 99^†^)	Group 3: Remote Canadian (n = 165^‡^)	p values		
				
				Group 1 vs 2	Group 1 vs 3	Group 2 vs 3
**Peripheral neuropathy**	10 (28%)**	9 (9%)	68 (47%)	0.007	0.041	< 0.001
**Abnormal 10 g monofilament**	7 (19%)	8 (8%)	22 (15%)	0.067	0.566	0.091
**Peripheral Vascular Disease**	2 (5.4%)	12 (12%)	16 (11%)	0.334	0.418	0.750
**Current foot ulcer**	1 (3%)	6 (6%)	7 (5%)	0.447	0.578	0.702
**Lower Extremity Amputation**	0	2 (2%)	0	0.390	-	0.087
**Retinopathy**	2 (7%)	18 (22%)	31 (24%)	0.070	0.202	0.615
**Microalbuminuria**^††^	18 (50%)	24 (25%)	67 (41%)	0.001	0.098	0.007
**Macroalbuminuria^‡‡^**	8 (22%)	12 (13%)	23 (14%)	0.012	0.065	0.298

Data are n (%).

* n = 32 for PVD, 32 for neuropathy, 29 for retinopathy. Note methods differed for retinopathy (Table 1).

^† ^n = 82 for retinopathy

**^‡^**n = 146 for neuropathy, 145 PVD, 142 foot ulcer, 144 amputation, 124 retinopathy.

**When definition of neuropathy in Group 1 is revised to be that of Group 2 then neuropathy in Group 1 is 19% (n = 6). Note methods differed for neuropathy in Group 3 (Table 1).

^††^Comparison across groups excludes those with macroalbuminuria

**^‡‡^**Comparison across groups excludes those with microalbuminuria

**Table 5:** Logistic regression of albuminuria (ACR ≥ 2.5 mg/mmol in men, ≥ 3.5 mg/mmol in women)

	Odds Ratio	95% Confidence Interval
**Urban Australian**	1.00	-
**Remote Australian**	7.24	2.60 - 20.23
**Remote Canadian**	3.15	1.66 - 6.01
**Systolic BP (mmHg)**	1.05	1.03 - 1.06
**HbA1c (%)**	1.13	1.00 - 1.29
Diabetes Duration* (years)	1.76	1.27 - 2.45

n = 268

*log transformed

The following variables were considered for inclusion in the final model: age, gender, weight, BMI, waist, current smoker, HbA1c, total cholesterol, triglycerides, diabetes duration.

## Discussion

Systematic reviews have drawn attention to the high prevalence of diabetes, CVD and CKD among Indigenous populations globally but studies from detailed clinical examination of participants are limited, and much of the available data stems from medical records or administrative databases [6,7]. This is an important gap because syntheses of detailed clinical data from even small cohorts may enhance our understanding of the extent and nature of these conditions. We have reported that although these Indigenous cohorts with diabetes from Australia and Canada displayed similarities for some key factors, there were significant differences for both CVD risk factors and microvascular complications of diabetes between groups. Rates of some CVD risk factors were higher in remote Indigenous Australians - central obesity (despite a lower BMI), dyslipidemia and CRP. Although rates of retinopathy and neuropathy were similar across the three groups, rates of nephropathy were higher in both remote groups than in the urban Australian group.

The three population groups reveal similarly common anthropometric, metabolic and lifestyle factors such as young age, mean HbA1c (8-8.7% across the groups), duration of diabetes (median duration 6 to 9 years across groups) and high rates of cigarette smoking (particularly in both remote groups with 50% current smokers). Consistent with previous reports, obesity and related comorbidities were also very common in all groups [10,26]. Interestingly, all 3 groups displayed relatively well controlled blood pressure, with mean values within each group within the targets of systolic < 130 and diastolic < 80 mmHg. Medication use was similar across the studies, with the exception of insulin, where use was lower in the remote Australian group, and metformin which was used more frequently in both Australian groups than in the Canadian group.

When compared to population-based data for the overall Australian population (from the AusDiab Study), achievement of therapeutic targets by urban Indigenous Australian participants with diabetes from the DRUID study was better for blood pressure and total cholesterol, but worse for glycemic control [15]. Although poor, the proportion of DRUID participants meeting glycemic therapeutic targets (HbA1c < 7%) was similar to that reported in secondary care settings in Australia [27,28], as well as in remote Northern Australian communities [29,30]. Possible contributory factors to the lower rate of achieving therapeutic targets for glycemic control (compared to good rates of blood pressure control) in the Indigenous populations of the current study include unmeasured factors such as psychosocial stress, socio-economic disadvantage and access to medical care, including access to diabetes education and support of self-management in the use of insulin. It is possible that more intensive use of insulin therapy could result in improved glycemic control in these populations [31], but an associated increase in culturally-appropriate diabetes education and support would need to be a key component of that management strategy. It is notable that rates of angiotensin converting enzyme inhibitor use were high across all 3 groups in the current study, although use of HMG CoA reductase inhibitors was only moderate. Rates of use of both of these classes of medications were higher in the current study than in the Aboriginal participants of the Fremantle Diabetes Study [32], however that study was performed a decade prior to the current studies. The greater use of angiotension converting enzyme inhibitors than of lipid-lowering medications in the current study may relate to the higher rates of albuminuria than elevated LDL-cholesterol in participants of the current study.

We reported a gradient of CVD and metabolic risk factors across the three groups with respect to central obesity, dyslipidemia and inflammatory markers with the most abnormal values being among remote Australians. The greater risk seen in remote compared to urban Indigenous Australians is consistent with previous reports relating to both CVD and end-stage kidney disease (ESKD). Cass et al. described a 20-30 fold gradient in rates of ESKD in Indigenous Australians across different regions of Australia [33], with higher rates in remote than urban regions, associated with greater socioeconomic disadvantage in remote communities [34]. The high levels of CRP in remote Indigenous Australians is consistent with previous reports^30, 31^; similar findings of higher inflammatory markers in urban India were postulated to relate to high rates of socioeconomic disadvantage [35-37]. Although CRP levels were lower in remote Canadians than both Australian groups, mean CRP was relatively high in all three groups. We have reported that CRP levels were independently elevated in participants with diabetes from the remote Canadian study, with no difference in CRP levels between carriers and non-carriers of the private polymorphism associated with increased prevalence of diabetes in that population (HNF1A G319S) in participants with diabetes [38]. We have previously reported very high fibrinogen levels among urban Indigenous Australian participants of the current study, and that fibrinogen levels were independently associated with CRP and HbA1c in that group [39]. There were also differences in body build and body composition across these population groups, with the striking difference in anthropometric indices for remote Indigenous Australians relating to lack of peripheral adiposity, demonstrated by significantly lower hip circumferences than both urban Australians and remote Canadians. To our knowledge, there are no published data comparing Indigenous Australian and Canadian communities for diabetes complications and CVD risk factors, although there have been previous reports relating to ESKD and HbA1c in these populations [40,41].

A striking finding of the current study relates to albuminuria - an exception to the above described gradient of risk from remote Indigenous Canadians to urban Indigenous Australians to remote Indigenous Australians. Rates of albuminuria were higher in both remote groups compared to the urban Australian group, with risk of albuminuria remaining independently higher in both remote groups after adjustment for other risk factors. This is consistent with previous reports of high rates of ESKD among Indigenous Australians and Canadians and the remote-urban gradient for ESKD among Indigenous Australians [34]. Of note, there were methodological differences between the studies for ACR, which was measured at the point-of-care in the Canadian study but in the laboratory in the Australian studies, however the remote Australian study did report good agreement between point-of-care and laboratory methods, consistent with previous reports [24,42].

Rates of microvascular complications other than albuminuria were similar across the three groups. There was no difference in neuropathy (by 10 gm monofilament), PVD or retinopathy rates. The trend to lower retinopathy rates among remote Australians could be explained by methodological differences (data were collected by chart review in that study compared to the use of retinal photography in other groups). There was a difference between the Australian groups in that a lower rate of wearing footwear was evident in the remote compared to the urban group, perhaps reflecting the relatively recent European contact for the remote group and different lifestyles between these groups. However rates of current foot ulcers did not differ between the three groups. The similar rates of microvascular complications of retinopathy and peripheral vascular disease across the three population groups may relate to similarities in HbA1c, but CVD risk factors show prominent differences between population groups. These differences may translate to different CVD outcomes and thus further study is required.

A limitation of this study is that the relatively small sample size of the cohorts which represent three distinct communities within Australia and Canada. It is fully appreciated that although there are many similarities in metabolic and CVD risks described here to those previously reported for other Indigenous groups within Australia and Canada, the results of this study may not reflect those of the general Indigenous populations of Australia and Canada. The urban Indigenous group were a volunteer cohort who represent only 14% of the target population, thus we are unable to comment as to whether they are representative of this population. Nevertheless, the DRUID Study is the largest and most comprehensive dataset on diabetes and related conditions in an urban Indigenous population in Australia. This is of importance as 73% of the Indigenous Australian population live in urban centres [43]. There is a similar paucity of data among urban Canadian Aboriginal people for diabetes complications, thus the Australian data may be of international importance. There were methodological differences between the three studies, particularly for assessment of blood pressure, neuropathy, retinopathy and biochemical variables. We have not assessed genetic factors, socioeconomic disadvantage, psychosocial stress or access to medical care. These factors likely play a role in the urban-remote differences reported between the Australian groups: the remote Australian group had less genetic admixture than the urban Australian group and although Indigenous Australians overall have lower incomes than non-Indigenous Australians, incomes decrease with increasing remoteness [1]. A recent review highlighted that even in countries where a universal health system is in place (such as Australia and Canada), socioeconomic and ethnic inequalities have been reported in the provision of health-care to those with diabetes [44]. Although health services are more likely to exist in urban than remote Australia, there may still be important barriers for Indigenous Australians accessing these services, as evident by reported delays for Indigenous Australians accessing thrombolysis to a similar extent in both urban and remote settings (compared to non-Indigenous Australians) [45]. However the strength of this report is the comprehensive clinical assessment of CVD risks and diabetes complications using standardized techniques, which was a feature of all three studies.

## Conclusions

Despite similarly high rates of diabetes, associated complications and premature CVD in these Indigenous populations, several key CVD risk factors were higher in the remote Australian group (central obesity, dyslipidemia, CRP) while albuminuria was notable for higher rates in both remote Indigenous Australians and Canadians than urban Indigenous Australians. In order to address the increased risk of diabetes and CVD in Indigenous communities, action is required from a young age both to prevent diabetes, and to improve management once it is established. As there are important differences in risk profile between Indigenous populations, including within the one country, this action needs to be designed and performed in partnership with communities and tailored to both community and individual needs.

## Competing interests

The authors declare that they have no competing interests.

## Authors' contributions

KOD and LMB designed and conducted the remote Australian study, JC, JS, KOD and LMB designed and conducted the DRUID study. AJH, SBH, MM, PC and BZ designed and conducted the Canadian study. LMB designed and performed the statistical analyses guided by AH, JC and KOD. LMB drafted the manuscript and all authors contributed important intellectual content. All authors approved the final version for publication. All authors read and approved the final manuscript.

## References

[B1] Australian Institute of Health and Welfare. The health and welfare of Australia's Aboriginal and Torres Strait Islander people, an overview 2011. Cat no: IHW 42 2011 Canberra: AIHW

[B2] Vos T Barker B Begg S Stanley L Lopez AD Burden of disease and injury in Aboriginal and Torres Strait Islander Peoples: the Indigenous health gap Int J Epidemiol 2009 38 2 470 477 19047078 10.1093/ije/dyn240

[B3] Young TK Reading J Elias B O'Neil JD Type 2 diabetes mellitus in Canada's first nations: status of an epidemic in progress CMAJ 2000 163 5 561 566 11006768 PMC80466

[B4] Potential years of life lost at ages 25 to 74 among Status Indians, 1991 to 2001. Statistics Canada, Catalogue no. 82-003-XPE http://www.statcan.gc.ca/pub/82-003-x/2011001/article/11409-eng.pdf

[B5] Potential years of life lost at ages 25 to 74 among Metis and non-Status Indians, 1991 to 2001. Statistics Canada, Catalogue no 82-003-XPE http://www.statcan.gc.ca/pub/82-003-x/2011001/article/11408-eng.pdf

[B6] Yu CHY Zinman B Type 2 diabetes and impaired glucose tolerance in aboriginal populations: A global perspective Diabetes Res Clin Pract 2007 78 2 159 170 10.1016/j.diabres.2007.03.022 17493702

[B7] Naqshbandi M Harris SB Esler JG Antwi-Nsiah F Global complication rates of type 2 diabetes in Indigenous peoples: A comprehensive review Diabetes Res Clin Pract 2008 82 1 1 17 10.1016/j.diabres.2008.07.017 18768236

[B8] Hoy WE Mathews JD McCredie DA Pugsley DJ Hayhurst BG Rees M Kile E Walker KA Wang Z The multidimensional nature of renal disease: rates and associations of albuminuria in an Australian Aboriginal community Kidney Int 1998 54 4 1296 1304 10.1046/j.1523-1755.1998.00099.x 9767547

[B9] Yeates K Tonelli M Chronic kidney disease among Aboriginal people living in Canada Clin Nephrol 2010 74 Suppl 1 S57 S60 20979965 10.5414/cnp74s057

[B10] Yeates K Tonelli M Indigenous health: update on the impact of diabetes and chronic kidney disease Curr Opin Nephrol Hypertens 2006 15 6 588 592 10.1097/01.mnh.0000247495.54882.e4 17053472

[B11] Katzenellenbogen JM Sanfilippo FM Hobbs MS Briffa TG Ridout SC Knuiman MW Dimer L Taylor KP Thompson PL Thompson SC Incidence of and case fatality following acute myocardial infarction in Aboriginal and non-Aboriginal Western Australians (2000-2004): a linked data study Heart Lung Circ 2010 19 12 717 725 10.1016/j.hlc.2010.08.009 20864399

[B12] Shah BR Hux JE Zinman B Increasing rates of ischemic heart disease in the native population of Ontario, Canada Arch Intern Med 2000 160 12 1862 1866 10.1001/archinte.160.12.1862 10871982

[B13] Fearnley E Li SQ Guthridge S Trends in chronic disease mortality in the Northern Territory Aboriginal population, 1997-2004: using underlying and multiple causes of death Aust N Z J Public Health 2009 33 6 551 555 10.1111/j.1753-6405.2009.00452.x 20078573

[B14] Maple-Brown LJ Brimblecombe J Chisholm D O'Dea K Diabetes care and complications in a remote primary health care setting Diabetes Res Clin Pract 2004 64 2 77 83 10.1016/j.diabres.2003.10.008 15063599

[B15] Maple-Brown L Cunningham J Dunne K Whitbread C Howard D Weeramanthri T Tatipata S Dunbar T Harper CA Taylor HR Complications of diabetes in urban Indigenous Australians: the DRUID study Diabetes Res Clin Pract 2008 80 3 455 462 10.1016/j.diabres.2008.01.011 18294723

[B16] Hanley AJG Harris SB Mamakeesick M Goodwin K Fiddler E Hegele RA Spence JD House AA Brown E Schoales B Complications of Type 2 Diabetes Among Aboriginal Canadians: Prevalence and associated risk factors Diabetes Care 2005 28 8 2054 2057 10.2337/diacare.28.8.2054 16043760

[B17] Brimblecombe J Mackerras D Garnggulkpuy J Maypilama E Bundhala L Dhurrkay R Fitz J Maple-Brown L Shemesh T Rowley KG Leanness and type 2 diabetes in a population of indigenous Australians Diabetes Res Clin Pract 2006 72 1 93 99 10.1016/j.diabres.2005.09.014 16260061

[B18] Cunningham J O'Dea K Dunbar T Weeramanthri TS Zimmet P Shaw J Study Protocol - Diabetes and related conditions in urban Indigenous people in the Darwin, Australia region: aims, methods and participation in the DRUID Study BMC Public Health 2006 6 8 10.1186/1471-2458-6-8 16417641 PMC1373687

[B19] Hanley A Harris SB Mamakeesick M Goodwin K Fiddler E Hegele RA McLaughlin JR Zinman B Complications of Type 2 Diabetes Among Aboriginal Canadians: Increasing the Understanding of Prevalence and Risk Factors Canadian Journal of Diabetes 2003 27 4 455 463

[B20] Cunningham J Diversity of primary health care providers for urban indigenous Australians Aust N Z J Public Health 2006 30 6 580 581 17211933 10.1111/j.1467-842x.2006.tb00793.x

[B21] Hanley AJG Harris SB Barnie A Gittelsohn J Wolever TMS Logan A Zinman B The Sandy Lake Health and Diabetes Project: Design, Methods and Lessons Learned Chronic Dis Can 1995 16 4 149 156

[B22] Waldram J Herring DA Young TK Aboriginal Health in Canada: historical, cultural, and epidemiological perspectives 1995 Toronto: University of Toronto Press

[B23] World Health Organisation. Definition, Diagnosis and Classification of Diabetes Mellitus and Its Complications 1999 Geneva: Department of Noncommunicable Disease Surveillance, WHO

[B24] Parsons MP Newman DJ Newall RG Price CP Validation of a point-of-care assay for the urinary albumin:creatinine ratio Clin Chem 1999 45 3 414 417 10053047

[B25] Tapp RJ Shaw JE Zimmet PZ Balkau B Chadban SJ Tonkin AM Welborn TA Atkins RC Albuminuria is evident in the early stages of diabetes onset: results from the Australian Diabetes, Obesity, and Lifestyle Study (AusDiab) Am J Kidney Dis 2004 44 5 792 798 15492944

[B26] O'Connell J Yi R Wilson C Manson SM Acton KJ Racial Disparities in Health Status Diabetes Care 2010 33 7 1463 1470 10.2337/dc09-1652 20357367 PMC2890342

[B27] Bryant W Greenfield JR Chisholm DJ Campbell LV Diabetes guidelines: easier to preach than to practise? Med J Aust 2006 185 6 305 309 16999670 10.5694/j.1326-5377.2006.tb00583.x

[B28] Flack JR Colagiuri S Final report of the Australian National Diabetes Information Audit and Benchmarking (ANDIAB) 2004 http://www.health.gov.au/internet/wcms/publishing.nsf/Content/pq-diabetes-pubs-andiab04 (Accessed April 2007) 10.1111/dme.13635 29633347

[B29] Bailie RS Si D Robinson GW Togni SJ D'Abbs PH A multifaceted health-service intervention in remote Aboriginal communities: 3-year follow-up of the impact on diabetes care Med J Aust 2004 181 4 195 200 15310253 10.5694/j.1326-5377.2004.tb06235.x

[B30] McDermott RA Tulip F Schmidt B Diabetes care in remote northern Australian Indigenous communities Med J Aust 2004 180 10 512 516 15139828 10.5694/j.1326-5377.2004.tb06055.x

[B31] Davis TM Davis WA Bruce DG Glycaemic levels triggering intensification of therapy in type 2 diabetes in the community: the Fremantle Diabetes Study Med J Aust 2006 184 7 325 328 16584365 10.5694/j.1326-5377.2006.tb00264.x

[B32] Davis TME McAullay D Davis WA Bruce DG Characteristics and outcome of type 2 diabetes in urban Aboriginal people: the Fremantle Diabetes Study Intern Med J 2007 37 1 59 63 10.1111/j.1445-5994.2006.01247.x 17199846

[B33] Cass A Cunningham J Snelling P Wang Z Hoy W End-stage renal disease in indigenous Australians: a disease of disadvantage Ethn Dis 2002 12 3 373 378 12148708

[B34] Cass A Cunningham J Wang Z Hoy W Regional variation in the incidence of end-stage renal disease in Indigenous Australians Med J Aust 2001 175 1 24 27 11476198 10.5694/j.1326-5377.2001.tb143507.x

[B35] McDonald S Maguire G Duarte N Wang XL Hoy W C-reactive protein, cardiovascular risk, and renal disease in a remote Australian Aboriginal community Clin Sci (Lond) 2004 106 2 121 128 10.1042/CS20030186 12956621

[B36] Rowley K Walker KZ Cohen J Jenkins AJ O'Neal D Su Q Best JD O'Dea K Inflammation and vascular endothelial activation in an Aboriginal population: relationships to coronary disease risk factors and nutritional markers Med J Aust 2003 178 10 495 500 12741936 10.5694/j.1326-5377.2003.tb05324.x

[B37] Yudkin J Yajnik C Mohamed-Ali V Bulmer K High levels of circulating proinflammatory cytokines and leptin in urban, but not rural, Indians. A potential explanation for increased risk of diabetes and coronary heart disease Diabetes Care 1999 22 2 363 364 10.2337/diacare.22.2.363 10333961

[B38] Ley SH Hegele RA Connelly PW Harris SB Mamakeesick M Cao H Gittelsohn J Retnakaran R Zinman B Hanley AJ Assessing the association of the HNF1A G319S variant with C-reactive protein in Aboriginal Canadians: a population-based epidemiological study Cardiovasc Diabetol 2010 9 39 10.1186/1475-2840-9-39 20716378 PMC2929219

[B39] Maple-Brown LJ Cunningham J Nandi N Hodge A O'Dea K Fibrinogen and associated risk factors in a high-risk population: urban Indigenous Australians, the DRUID Study Cardiovasc Diabetol 2010 9 69 10.1186/1475-2840-9-69 21029470 PMC2988000

[B40] Yeates KE Cass A Sequist TD McDonald SP Jardine MJ Trpeski L Ayanian JZ Indigenous people in Australia, Canada, New Zealand and the United States are less likely to receive renal transplantation Kidney Int 2009 76 6 659 664 10.1038/ki.2009.236 19553910

[B41] Daniel M O'Dea K Rowley KG McDermott R Kelly S Glycated hemoglobin as an indicator of social environmental stress among indigenous versus westernized populations Prev Med 1999 29 5 405 413 10.1006/pmed.1999.0559 10564632

[B42] Shemesh T Rowley KG Shephard M Piers LS O'Dea K Agreement between laboratory results and on-site pathology testing using Bayer DCA2000+ and Cholestech LDX point-of-care methods in remote Australian Aboriginal communities Clin Chim Acta 2006 367 1-2 69 76 10.1016/j.cca.2005.11.014 16388790

[B43] ABS & AIHW (Australian Bureau of Statistics & Australian Institute of Health and Welfare). The Health and Welfare of Australia's Aboriginal and Torres Strait Islander Peoples Canberra 2005

[B44] Ricci-Cabello I Ruiz-Pérez I De Labry-Lima AO Márquez-Calderón S Do social inequalities exist in terms of the prevention, diagnosis, treatment, control and monitoring of diabetes? A systematic review Health Soc Care Community 2010 18 6 572 587 10.1111/j.1365-2524.2010.00960.x 21040063

[B45] Ong MA Weeramanthri TS Delay times and management of acute myocardial infarction in indigenous and non-indigenous people in the Northern Territory Med J Aust 2000 173 4 201 204 11008594 10.5694/j.1326-5377.2000.tb125601.x

